# Identification of hsa-miR-619-5p and hsa-miR-4454 in plasma-derived exosomes as a potential biomarker for lung adenocarcinoma

**DOI:** 10.3389/fgene.2023.1138230

**Published:** 2023-05-11

**Authors:** Linxiang Feng, Zian Feng, Jie Hu, Jiahui Gao, Ang Li, Xiaodong He, Liu Liu, Zuojun Shen

**Affiliations:** ^1^ Department of Laboratory Medicine, The First Affiliated Hospital of USTC, Division of Life Sciences and Medicine, University of Science and Technology of China, Hefei, Anhui, China; ^2^ Core Unit of National Clinical Research Center for Laboratory Medicine, Hefei, China

**Keywords:** lung adenocarcinoma, exosome, miRNA, bioinformatics, qRT-PCR

## Abstract

**Introduction:** Lung cancer has long been at the forefront of all cancers in terms of incidence and mortality. Lung adenocarcinoma is the most common type of lung cancer, accounting for 40% of all lung cancer types. Exosomes can act as biomarkers of tumors and thus play an important role.

**Methods:** In this article, high-throughput sequencing of miRNAs in plasma exosomes from lung adenocarcinoma patients and healthy individuals was performed to obtain 87 upregulated miRNAs, which were then combined with data from the GSE137140 database uploaded by others for screening. The database included 1566 preoperative lung cancer patients, 180 postoperative patients, and 1774 non-cancerous controls. We overlapped the miRNAs upregulated in the serum of lung cancer patients in the database relative to those of non-cancer controls and post-operative patients with the upregulated miRNAs obtained from our next-generation sequencing to obtain nine miRNAs. Two miRNAs that were not reported as tumor markers in lung cancer, hsa-miR-4454 and hsa-miR-619-5p, were selected from them and then validated by qRT-PCR, and further analysis of miRNAs was performed using bioinformatics.

**Results:** Real-time quantitative PCR showed that the expression levels of hsa-miR-4454 and hsa-miR-619-5p in plasma exosomes of patients with lung adenocarcinoma were significantly up-regulated. The AUC values of hsa-miR-619-5p and hsa-miR-4454 were 0.906 and 0.975, respectively, both greater than 0.5, showing good performance. The target genes of miRNAs were screened by bioinformatics methods, and the regulatory network between miRNAs and lncRNAs and mRNAs was studied.

**Discussion:** Our work demonstrated that hsa-miR-4454 and hsa-miR-619-5p have the potential to be used as biomarkers for the early diagnosis of lung adenocarcinoma.

## 1 Introduction

Lung cancer is one of the major malignant tumors that endanger human health. With the development of medical technology, the incidence and mortality rate of lung cancer has decreased, but it is still the second most prevalent malignancy and the first mortality rate in the world (Siegel et al., 2022). Lung cancer can be classified into two main types by pathology: small cell lung cancer and non-small cell lung cancer. Non-small cell lung cancer is the more prevalent type, accounting for approximately 85% of all lung cancer cases (Chen et al., 2021). In contrast, lung adenocarcinoma (LUAD) is the most common non-small cell lung cancer subtype, accounting for 40% of all lung cancer types and reaching about 55% of non-small cell lung cancers (Yao et al., 2021). Small cell lung cancer has a very insidious onset and most are not diagnosed until the middle to late stages, with a low 5-year survival rate (Yao et al., 2021; Miller et al., 2022). Currently used for clinical screening of lung adenocarcinoma tumor markers with carcinoembryonic antigen (CEA), cytokeratin-19 fragment (CYFRA21-1) and neuron-specific enolase (NSE) and so on, but these protein markers are prone to false positive, and their specificity and sensitivity are not high (Yuan et al., 2022). Therefore, the search for novel tumor markers with rapid non-invasive, high specificity, and sensitivity for non-small cell lung cancer are of utmost importance.

Liquid biopsy is the process of obtaining tumor-derived material such as tumor DNA, RNA, intact tumor cells, extracellular vesicles, and tumor-inducing platelets from body fluids such as blood, urine, saliva, feces, and even cerebrospinal fluid (Li et al., 2020). Its relatively non-invasive nature compared to conventional tumor tissue biopsy makes it an attractive modality for research (Chen and Zhao, 2019). The main tests currently available for tumor liquid biopsy techniques include CTCs (circulating tumor cells), ctDNA (DNA released by tumor cells into peripheral blood), and exosomes (Ye et al., 2019). They can be used for early screening of cancer, real-time monitoring of cancer, determining cancer prognosis, and acting as transport carriers (Chen and Zhao, 2019; Ye et al., 2019). Exosomes are double-layered lipid-coated extracellular vesicles produced by living cells and are approximately 50–150 nm in diameter, usually in the form of disks, spheres, etc. (Huang et al., 2019; Pegtel and Gould, 2019). Exosomes are produced as a result of cellular endocytosis and carry a variety of molecules for eventual secretion outside the cell (Zhang and Yu, 2019). When exosomes are taken up by recipient cells, they transfer miRNAs, mRNAs, proteins, lipids, and other substances, which can alter the molecular composition of the recipient cells. This process can affect the function of the recipient cells through gene regulation and signaling pathways (Zhang and Yu, 2019). Studies have shown that exosomes influence the biological functions of a wide range of cells in the extracellular microenvironment through immune regulation, morphogenetic signaling, delivery of genetic material, and cellular recruitment (Gurunathan et al., 2019).

microRNAs (miRNAs) are a class of small, highly conserved, endogenous non-coding, RNAs that are approximately 20–25 nucleotides in length. They are often clustered in introns of protein-coding genes and are primarily found in eukaryotes, (Ali Syeda et al., 2020; Hu et al., 2020). According to the literature, miRNAs play an important regulatory role in cell proliferation, apoptosis, invasion and metastasis by silencing specific genes through post-transcriptional inhibition of translation of target mRNAs during the development of a variety of malignancies (Bracken et al., 2016; Zhang B. et al., 2019; Wu et al., 2022). miRNAs are involved in regulating almost all cellular pathways associated with cancer, including differentiation, proliferation and survival (Zhang X. et al., 2019). Thus, abnormal expression of miRNAs may play a role in different types of cancer, including lung cancer (Wu et al., 2022). Elevated levels of many specific miRNAs lead to decreased expression of tumor suppressor genes, thereby increasing tumor growth and metastasis (Hayes et al., 2014; Bracken et al., 2016). miRNAs in exosomes have similar effects. Because the membranous vesicle structure of exosomes can protect miRNA from the decomposition of RNA enzyme and trypsin, it can exist stably in peripheral blood, which provides a basic guarantee for the biological function of exosome miRNA. Research confirms that exosomal miRNAs play a key role in altering the lung cancer microenvironment and may contribute to lung cancer progression, invasion, angiogenesis, metastasis, and drug resistance (Guiot et al., 2019).

In this project, we extracted exosomes from the plasma of lung adenocarcinoma patients and analyzed the differential expression of miRNAs from the level of exosomes, aiming to understand the relationship between exosomal miRNAs and lung adenocarcinoma and provide new biomarkers for early diagnosis of lung adenocarcinoma.

## 2 Materials and methods

### 2.1 Collection of patient samples

A total of 86 plasma samples, including 43 from healthy individuals and 43 from patients with lung adenocarcinoma, were collected at the First Hospital of the University of Science and Technology of China (Supplementary Table S1). All were collected in the morning on an empty stomach, without surgery or drug treatment. Upper plasma samples were collected in anticoagulant tubes containing EDTA anticoagulant and centrifuged at 1500g, 4°C for 10 min for subsequent experiments. Six of the samples were used for next-generation sequencing to screen miRNA and 80 samples were used for qRT-PCR verification.

### 2.2 Extraction of exosomes

Exosomes were extracted from plasma samples collected by SBI’s ExoQuick Plasma prep and Exosome precipitation kit (EXOQ5TM-1). 500 μL of plasma was absorbed, thrombin was added to remove fibrin, and the supernatant was collected and mixed with a proportion of ExoQuick. The supernatant was removed and resuspended in 500 μL PBS. And they refrigerated at −80°C for later use.

### 2.3 Identification of exosomes

Exosomes were identified using three classical methods. Antibodies used were CD63 (AB68418, abcam), TSG101 (AB125011, abcam), Calnexin (10427-2-AP, proteintech), and western blotting to detect whether the signature proteins of exosomes were expressed in exosomes. The secondary antibody was Anti-Rabbit IgG (H + L) HRP Conjugate (Promega). Samples were placed on copper grids and negatively stained with phosphotungstate, then the morphology of the extracted exosomes was observed by transmission electron microscopy (TEM). And the diameter of the exosomes was examined by nanoparticle tracking analysis (NTA). The above methods proved that we successfully extracted exosomes.

### 2.4 Extraction of exosomal RNA

The exosomal RNA was extracted using the TRIzol-chloroform method. After full lysis by adding TRIzol (ThermoFisher), an appropriate amount (1 pmol) of cel-miR-39 (Sohn et al., 2015) (RiboBio) was added, followed by chloroform (TRIzol: chloroform = 5:1) to stratify the RNA. The RNA was then washed twice with 75% ethanol, the supernatant was removed and the RNA was resuspended in 30 µL DEPC water. And then concentration was measured on an ultra-micro spectrophotometer (Nanodrop one) for subsequent experiment.

### 2.5 RNA-seq analysis

Transcriptome sequencing was performed on plasma exosomes from 3 lung cancer patients and 3 healthy controls. High throughput transcriptome sequencing using BGISEQ sequencing platform. In short, linear DNA amplification sequencing is performed using DNA nanospheres (DNB). According to the manufacturer’s instructions, a Unique molecular identifier (UMI) is introduced during library construction, which is attached to the cDNA molecule at the initial stage of library construction, and each molecule in the original sample is labeled. RNA libraries were quantified using Agilent 2,100 BioAnalyzer (Agilent Technologies). Finally, according to the amplification procedure, sequencing was performed using a combination of probe - anchored polymerization (cPAS).

### 2.6 Bioinformatic screening of miRNA

Plasma exosomal RNA was extracted from three lung adenocarcinoma patients and three healthy individuals, and the plasma exosomal miRNA expression profiles of lung adenocarcinoma were screened by next-generation sequencing. Sequencing data from lung adenocarcinoma patients were found in the GEO database, and the sequencing results from our laboratory were compared with the database results. Venn diagrams were used for screening differentially expressed miRNAs in lung adenocarcinoma patients. In the GSE137140 dataset, receiver operating characteristic (ROC) analysis was performed on the candidate upregulated miRNAs and their AUC (the area under the curve) values were calculated.

### 2.7 Reverse transcription and qRT-PCR of exosomal miRNAs

The sequences of hsa-miR-4454 and hsa-miR-619-5p were searched in miRbase (https://mirbase.org/) (Kozomara et al., 2019). Reverse transcription primers were then designed using the stem-loop method, which allows one gene to correspond to one specific primer. The extracted RNA is reversed using the PrimeScript™ RT reagent Kit (Takara). The system was 10 µL and the whole process was carried out on the ice, following conditions are set according to the instructions. Afterwards the forward and reverse primers were designed, and then conditions were set according to the TB Green^®^ Premix Ex Taq™ II (Takara) kit instructions, the system was 20 µL and the whole process was carried out on ice. The genes were amplified on the qRT-PCR instrument (Roche LightCycler 96). The primers were designed as shown in Supplementary Table S2.

### 2.8 Prediction and function of target genes

Two bioinformatics software including Targetscan (https://www.targetscan.org/vert_72/) and miRWalk (http://mirwalk.umm.uni-heidelberg.de/) were used to predict the target genes of hsa-miR-4454 and hsa-miR-619-5p (Agarwal et al., 2015; Sticht et al., 2018). And to get more accurate results, we took the overlap of the predictions of the two software for hsa-miR-4454 and hsa-miR-619-5p. Then target gene was ABHD2. And in lung cancer tissue sequencing samples (TCGA database), ABHD2 was tested for significant downregulation. And the AUC value of ABHD2 was calculated.

### 2.9 Data analysis

The data obtained by qRT-PCR were used to calculate the relative expression using 2^−ΔΔCT^ method, and finally, t-tests were performed and graphs were used GraphPad Prism 9.0. ROC curves were used to evaluate the diagnostic accuracy of candidate miRNAs and their combinations, and AUC was calculated. *p* < 0.05 and *p* < 0.001 were considered statistically significant. The relationship network diagram was constructed using Cytoscape 3.9.1.

## 3 Results

### 3.1 Identification of exosomes

**FIGURE 1:**
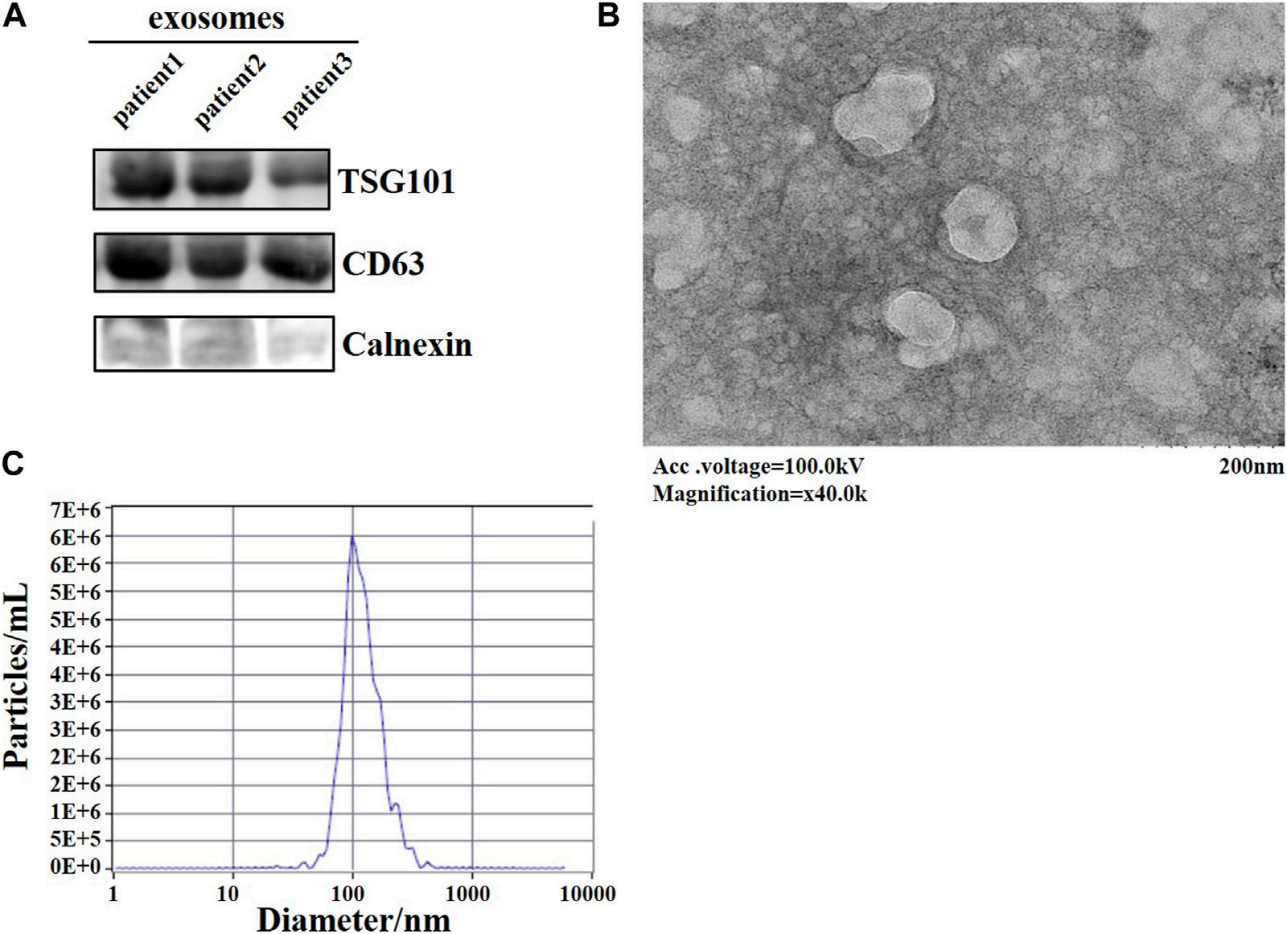
Identification of plasma-derived exosomes from lung adenocarcinoma patients. **(A)** WB assay to detect the expression of signature proteins of exosomes. **(B)** TEM observation of the morphology of exosomes. **(C)** The diameter of exosomes was examined by NTA.

After extracting exosomes from lung adenocarcinoma patients’ plasma, we used three methods to identify exosome. CD63, TSG101, which are enriched in exosomes, and Calnexin, which is not present in exosomes. Samples were subjected to western blotting, both TSG101 and CD63 were found to be expressed in exosomes, whereas Calnexin was hardly expressed in exosomes (Figure 1A). The morphology of the exosomes was observed under transmission electron microscopy as a spherical disc-like structure (Figure 1B). NTA showed the diameter of the exosome was around 100 nm (Figure 1C). Taken together, we have successfully extracted exosomes, which can be used for subsequent experimental studies.

### 3.2 Screening of candidate miRNAs

**FIGURE 2:**
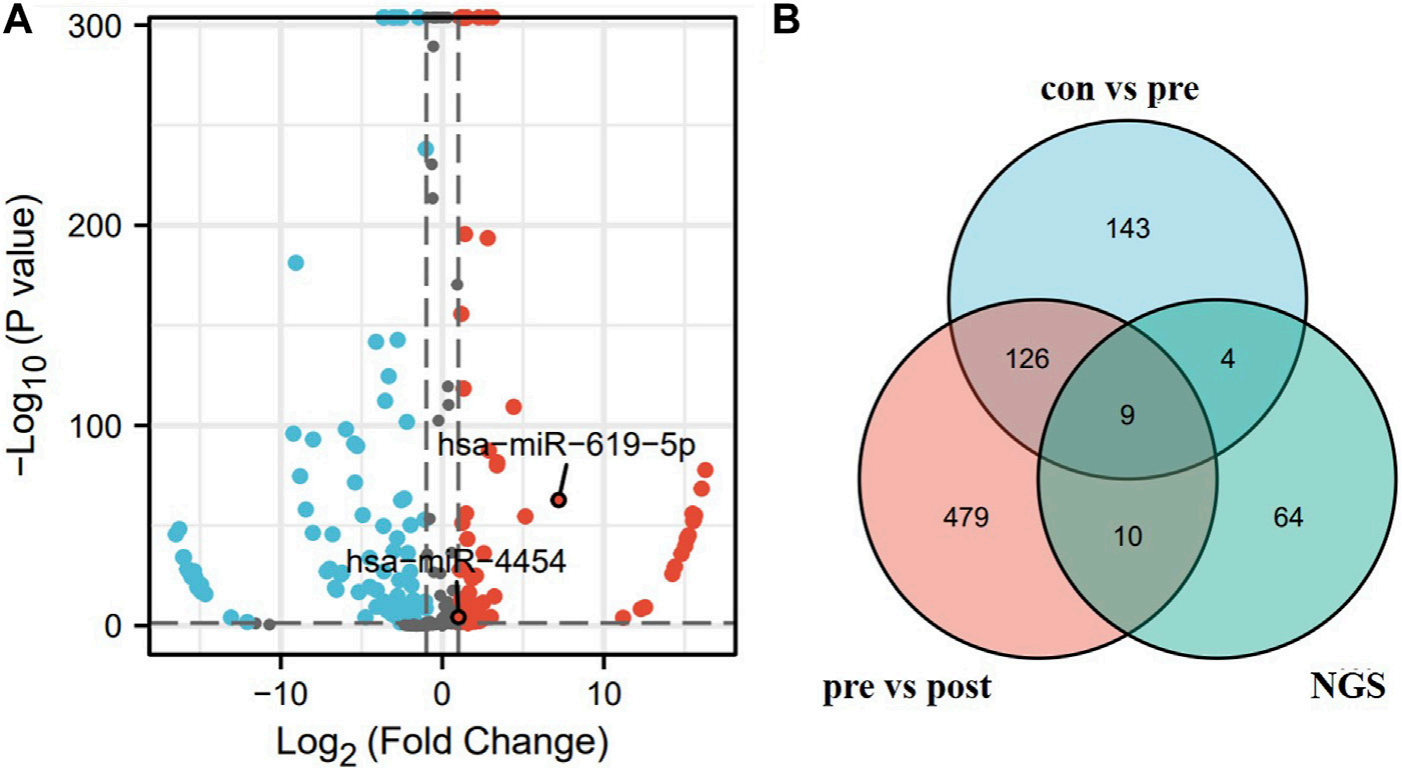
Screening of candidate miRNAs. **(A)** Volcano map of differential genes obtained by next-generation sequencing. **(B)** Blue area represents the miRNA upregulated in the serum of 1566 lung cancer patients *versus* 1774 healthy individuals in the GSE137140 database. Red area represents the miRNA upregulated in the serum of 1566 lung cancer patients *versus* 180 post-operative patients in the GSE137140 database, and green area represents the upregulated miRNA obtained from sequencing results.

We combined the differentially expressed genes from next-generation sequencing with bioinformatics to screen for miRNAs. Figure 2A showed the volcanoes of differentially expressed genes from the next-generation sequencing (BioProject ID: PRJNA699702) analysis, blue dots represent downregulated genes while red dots represent upregulated genes. Exosomal miRNAs were detected in three LUAD patients and three non-tumor controls, 87 miRNAs were upregulated in lung adenocarcinoma patients in differentially expressed miRNAs (DEM) analysis (|log2 [FC]|≥1, *p* < 0.05). Candidate miRNAs were screened using a combination of bioinformatics and next-generation sequencing. The GEO database contained 1566 lung cancer patients (preoperative patients), 1774 healthy individuals (control), and 180 post-operative patients. The three sets of data were compared in pairs (con vs. pre, pre vs. post), then intersects with the sequencing results, nine candidate genes were selected: hsa-miR-4454, hsa-miR-23b-3p, hsa-miR-140-3p, hsa-miR-151b, hsa-miR-320d, hsa-miR-619-5p, hsa-miR-28-5p, hsa-miR-19b-3p, and hsa-miR-17-5p, respectively (Figure 2B).

### 3.3 Verification of miRNA-seq data

**FIGURE 3:**
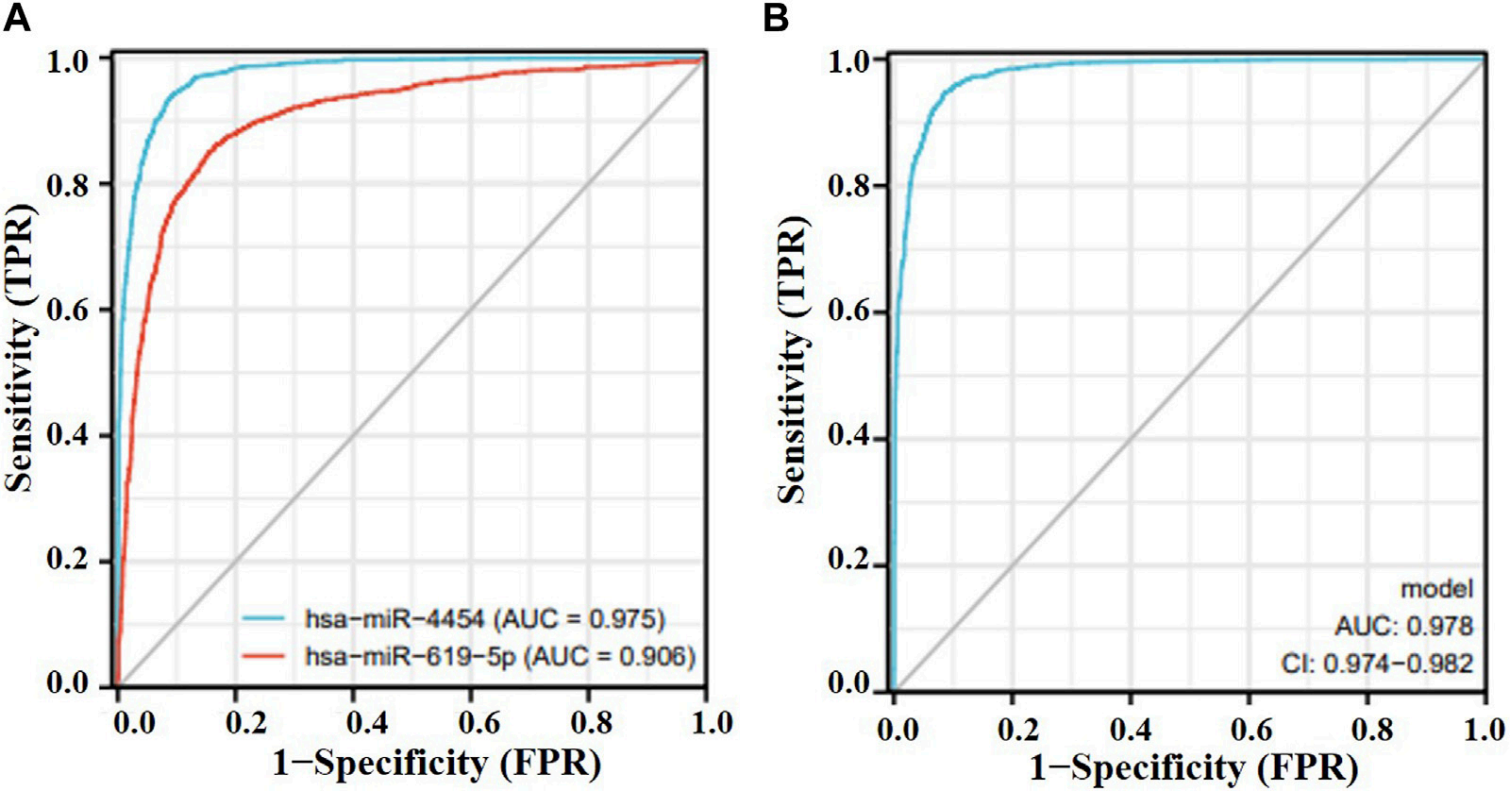
Effectiveness of miRNAs as biomarkers for lung cancer. **(A)** hsa-miR-619-5p; hsa-miR-4454. **(B)** hsa-miR-4454+hsa-miR-619-5p.

Among the nine differentially expressed candidate exosomal miRNAs, two novel miRNAs were selected for verification while highly expressed in lung adenocarcinoma. To demonstrate the performance of these two genes in lung cancer, the AUC values of hsa-miR-619-5p and hsa-miR-4454 were calculated to be 0.906 and 0.975, respectively, in the GSE137140 database (Figure 3A). We used a logistic model to integrate hsa-miR-4454 and hsa-miR-619-5p, the logistic regression model = −33.904 + 2.808*hsa-miR-4454 + 0.4687*hsa-miR-619-5p, which resulted in an AUC value of 0.978 (confidence interval: 0.974–0.982, *p* < 0.001) (Figure 3B). These results indicate that the specificity and sensitivity of hsa-miR-4454 and hsa-miR-619-5p are both high and suitable for further investigation. However, there was little difference between the specificity and sensitivity of the combination and that of hsa-miR-4454.

### 3.4 qRT-PCR verification

**FIGURE 4:**
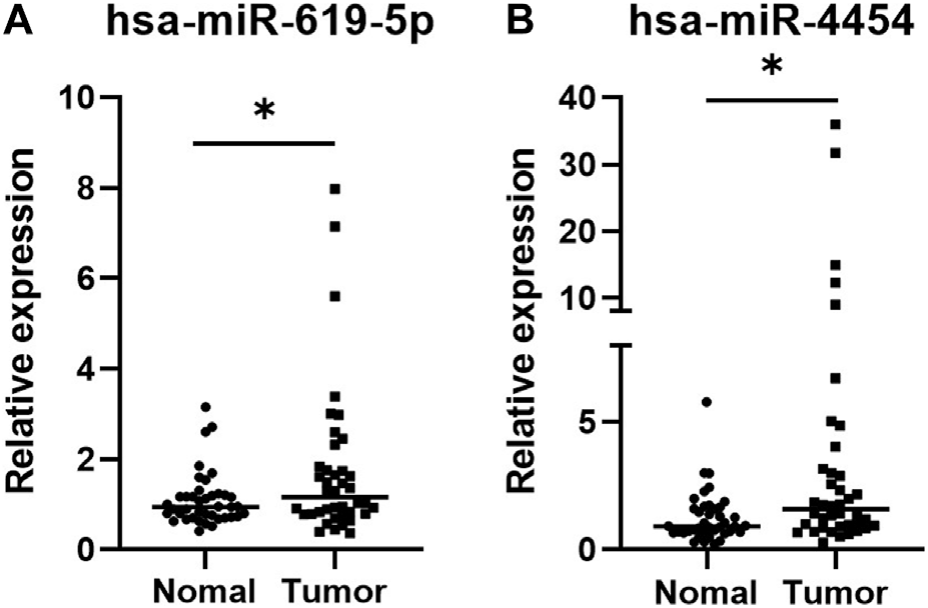
Expression levels of two miRNAs in plasma exosomes of 40 LUAD patients and 40 healthy individuals. The values of the relative gene expression were normalized to Cel-miR-39 and calculated using the 2^−ΔΔCT^ method. **(A)** hsa-miR-5p; **(B)** hsa-miR-4454. *p < 0.05.

qRT-PCR was applied to verify the significance of the sequencing results, 40 LUAD plasma exosome samples and 40 control samples were taken for the test. As shown in Figure 4, both hsa-miR-619-5p and hsa-miR-4454 were significantly upregulated in plasma exosomes from LUAD patients compared to controls (*p* < 0.05), which is consistent with previous sequencing results. The above qRT-PCR experiments verified the sequencing results, showing that these two genes have high clinical significance in the occurrence of lung cancer.

### 3.5 Study of target genes in lung cancer

**FIGURE 5:**
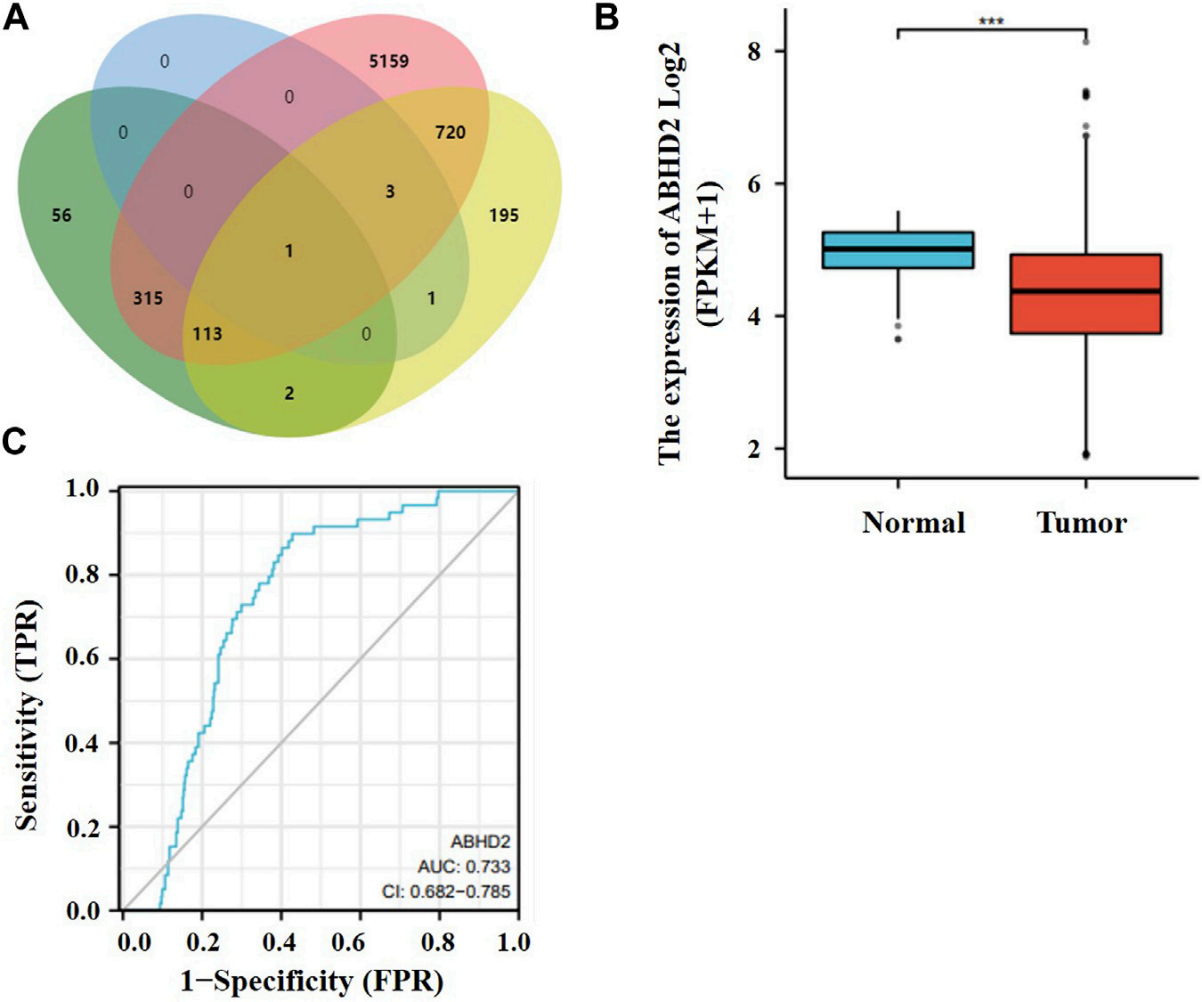
Target gene studies in lung cancer. **(A)** Prediction of downstream genes of hsa-miR-619-5p and hsa-miR-4454. Green and pink parts are downstream genes of hsa-miR-619-5p predicted in miRWalk and Targetscan respectively. Blue and yellow parts are downstream genes of hsa-miR-4454 predicted in miRWalk and Targetscan respectively. **(B)** ABHD2 was significantly downregulated in lung adenocarcinoma tissue samples. **(C)** AUC values of ABHD2 in lung adenocarcinoma. ***p < 0.001.

The target gene of these two miRNAs were predicted by bioinformatics method, and the role of the target gene in lung cancer was analyzed. By analyzing their downstream genes separately using two different software, after comparing the results, the common gene ABHD2 (Figure 5A) was found. ABHD2 was found to be significantly downregulated in lung adenocarcinoma tissue samples (TCGA database) from others (Figure 5B) and the AUC value of ABHD2 in lung adenocarcinoma was 0.733 (Figure 5C). Therefore, hsa-miR-619-5p and hsa-miR-4454 may regulate the occurrence and development of lung adenocarcinoma by regulating their downstream gene ABHD2.

### 3.6 The relationship between lncRNA, miRNA and mRNA

**FIGURE 6:**
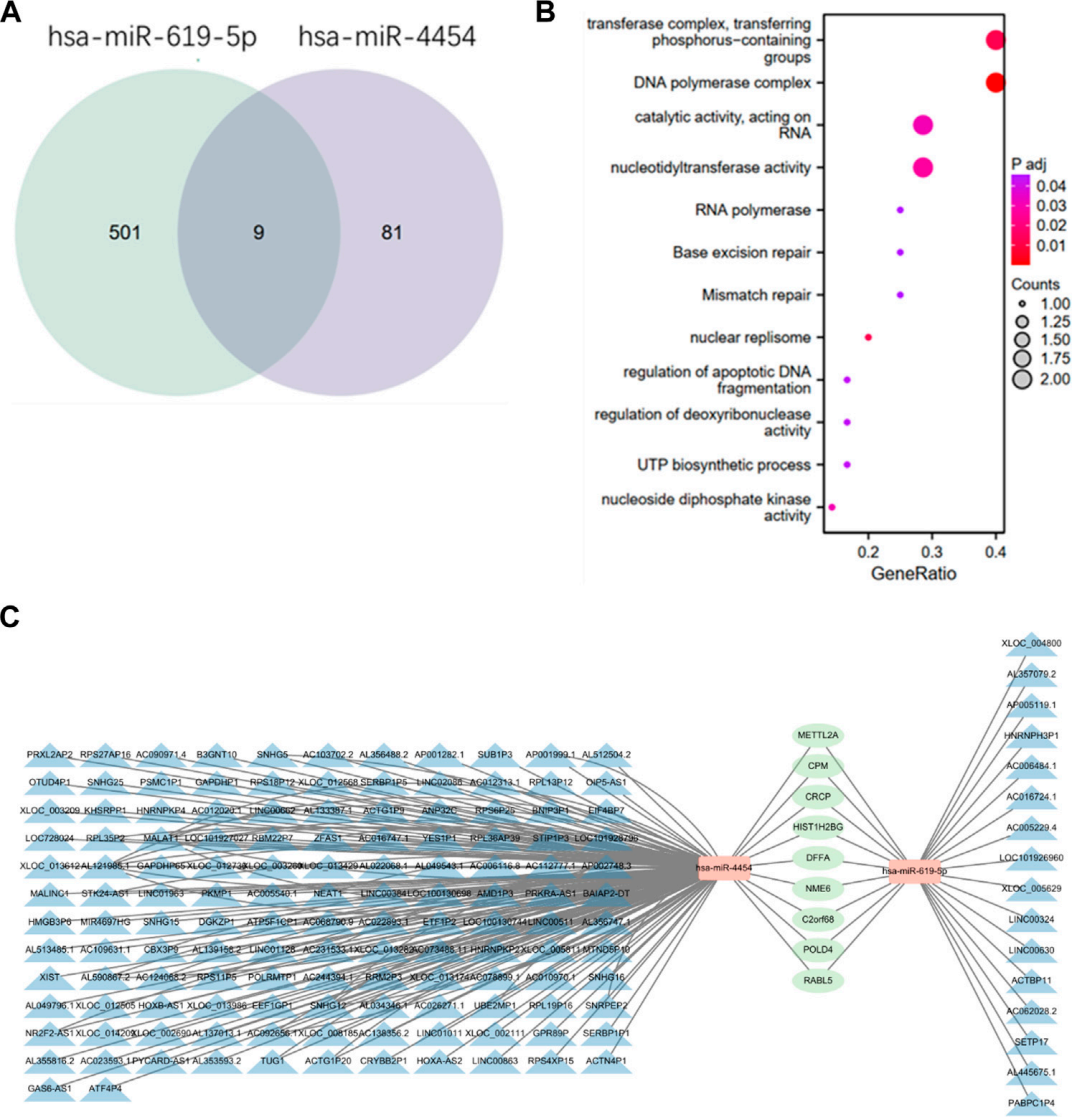
Relationship between two miRNAs, lncRNA and mRNA. **(A)** Prediction of the intersection of target genes obtained by two miRNAs by TargetScan online. **(B)** Functional enrichment of the obtained target genes. **(C)** The blue part is the lncRNAs related to the two miRNAs predicted by DIANA TOOLS online website, the orange part is miRNA, and the green part is the target genes of the two miRNAs. The relationship network diagram of the three parts is constructed using cytoscape software.

Many lncRNAs have a structure similar to mRNA, so miRNAs can negatively regulate lncRNAs through a mechanism similar to that of mRNA. Therefore, it is very important to study the relationship between miRNA, LncRNA and mRNA. We use DIANA TOOLS online website (http://diana.imis.athena-innovation.gr/DianaTools/index.php) to predict lncRNAs associated with these two miRNAs. Using TargetScan (https://www.targetscan.org/vert_80/) to predict target genes of the two miRNAs, and binding the resulting target genes to get nine target genes (Figure 6A). Then functional enrichment of these 9 target genes was performed by bioinformatics (Figure 6B). Cytoscape software is used to plot the network diagram of relations between the three (Figure 6C).

## 4 Discussion

In recent years, the diagnostic significance of exosomes in clinical diseases has been more widely studied. Exosomes are small vesicles enclosed in lipid molecular membranes that exist in a variety of body fluids and secreted by all cells. Plasma exosome testing is a reproducible and painlessmethod to conventional tissue biopsies. It can act as an indicator of disease progression and prognosis (Banys-Paluchowski et al., 2020). Compared with the traditional methods, the exosome extraction method used in this study has the advantages of simplicity, efficiency and rapidity, and does not have safety risks (Li et al., 2017). In this study, we used TEM, NTA, and western blotting to identify exosomes. Through TEM we visualized the typical structure of exosomes: a vesicle-like material with a cup-shaped structure. NTA detects the size distribution of particles dispersed in solution by the principle of light scattering and Brownian motion, the particle size of the extracted exosomes is mainly around 100 nm, which consistent with the expected result. Various intracellular proteins are involved in the formation and transport of exosomes (Wu and Shen, 2020). Among them, CD63 is a four-transmembrane protein directly involved in the sorting of exosomal contents; ALIX is directly involved in plasma membrane cleavage to form independent membrane vesicles; TSG101 is the driver of membrane formation and breakage; HSP70 and HSP90 are involved in the formation of multivesicular intracellular bodies, and these surface signature membrane proteins can be used as markers to identify exosomes (Lässer et al., 2012; Raposo and Stoorvogel, 2013; Zhao et al., 2021). In this study, two surface signature proteins, CD63 and TSG101, were selected to identify plasma exosomes, and the results showed that CD63 and TSG101 proteins were positively expressed in plasma exosomes, whereas Calnexin was not expressed in exosomes.

Based on the role of miRNA in disease diagnosis, we focused on the potential application of miRNAs in plasma exosomes as biomarkers for the diagnosis of lung adenocarcinoma. A large number of studies have reported the potential application value of miRNA as biomarkers in lung adenocarcinoma (Ghafouri-Fard et al., 2020; Nie et al., 2020; Wang et al., 2021). Exosomes as a new research hotspot in recent years, the role of exosomal miRNA has also received extensive attention. In this study, candidate miRNAs were obtained using sequencing results and online database screening, the specificity and sensitivity of hsa-miR-4454 and hsa-miR-619-5p in plasma exosomes from patients with early-stage lung adenocarcinoma were further reported. Tumor cells exchange essential information with other receptor cells by releasing exosomes carrying proteins, lipids and nucleic acids (Kalluri, 2016; Kalluri and LeBleu, 2020). The lipid membrane structure of exosomes protects their contents from degradation by RNases and trypsin, this can enhance the potential of exosomal miRNAs as diagnostic and prognostic biomarkers for cancer (Kalluri, 2016; Kalluri and LeBleu, 2020). In this project, we investigated the expression of hsa-miR-4454 and hsa-miR-619-5p in plasma exosomes from lung adenocarcinoma patients. The significance of these two novel miRNAs was verified by qRT-PCR analysis. Both of them are significantly expressed in various cancers according to previous studies, but none of the studies have been performed in plasma exosomes and were lack of clinical applications. Furthermore, the expression of these two genes in patients with lung adenocarcinoma and postoperative patients need to be further studied.

Next we focus on the specific mechanisms of these two miRNAs for cancer therapy to understand their subsequent impact on tumor biology.

Kim et al., in 2020 described that miR-619-5p is abundant in non-small cell lung cancer cells and targets RCAN1.4 to promote tumor angiogenesis and metastasis. Although there have been basic studies in lung cancer, it has not been reported in terms of its function as a potential diagnostic marker for lung adenocarcinoma (Kim et al., 2020). Qiu et al. found that MALAT1 and miR-619-5p have potential in the molecular diagnosis of patients with advanced colorectal cancer, and the combined detection of MALAT1 and miR-619-5p may improve the accuracy of colorectal cancer diagnosis and may serve as a good indicator of prognosis for patients with advanced colorectal cancer (Qiu et al., 2016). Gu et al. showed that LINC01485 could act as a ceRNA for miR-619-5p to affect RUNX2 expression and thus promote osteogenic differentiation of human bone marrow mesenchymal stem cells (hBMSCs). The LINC01485/miR-619-5p/RUNX2 axis may be a new target in the field of bone tissue engineering (Gu et al., 2022). Ma et al. also found that CIRC-FAT1 gene downregulation suppressed colorectal carcinogenesis by sponging miR-619-5p to inhibit FOSL2 expression, providing a potential pathway for colorectal cancer treatment (Ma et al., 2022). Wei Liu et al. in their report found that ILF3-AS1 inhibited colorectal carcinogenesis by regulating the miR-619-5p/CAMK1D axis in colon adenocarcinoma (COAD) plays an oncogenic role (Liu and Li, 2021). Mu et al. found that in glioma, reduced BCYRN1 expression was associated with poor patient prognosis. lncRNA BCYRN1 plays a ceRNA role by regulating CUEDC2 expression and PTEN/AKT/p21 pathway through sponge miR-619-5p, thereby inhibiting glioma development (Mu et al., 2020). The results of Chen et al. elucidated that lncRNA SBF2-AS1 promotes proliferation and invasion of colorectal cancer by suppressing miR-619-5p activity, providing new clues to the prognosis of lncRNA related to colorectal cancer (Chen et al., 2019). A study by Song et al. identified that miR-619-5p is involved in oral squamous cell carcinoma by regulating ATXN3. An important regulator involved in cisplatin resistance in oral squamous cell (OSCC) may serve as a potential therapeutic target (Song et al., 2021). From the above studies on hsa-miR-619-5p, it can be found that it is mainly an important link in the middle of the mechanistic studies of cancer therapy, mainly by targeting its target genes for regulatory purposes. The upstream generally regulates the expression of downstream proteins or pathways through lncRNA or circRNA.

In a study of miR-4454, Kannathasan et al. found that miR-4454 as a microRNA-based treatment to silence GNL3L (a key regulator of NF-κB signaling) may significantly reduce oncogenic GNL3L/NF-kappa B signaling-dependent cell survival, making miR-4454 a candidate for the treatment of metastatic human colorectal cancer (Kannathasan et al., 2020). A study by Lin et al. studied that miR-4454 promoted hepatocellular carcinoma progression by targeting Vps4A and Rab27A, while miR-4454 inhibitor and miR-4454 inhibitor-mediated exosomes significantly inhibited hepatocellular carcinoma cell proliferation, migration invasion and angiogenesis and accelerated cycle arrest, apoptosis and ROS(Lin et al., 2021). Wang et al. found that miR-4454 promoted proliferation, invasion and migration and inhibited apoptosis of cervical cancer cells by targeting ABHD2/NUDT21 (Wang et al., 2020). Dasari et al. found that miR-4454 promotes migration, invasion, proliferation and clonal growth of ovarian cancer cells in metastatic ovarian cancer cells through downregulation of paracrine signaling in the microenvironment. Since microenvironment-induced miR-4454 downregulation is critical for early and advanced metastasis, targeting miR-4454 may be a promising therapeutic approach (Dasari et al., 2020). The above studies suggest that miR-4454 is a promising target molecule for cancer therapy, mainly through targeting target genes involved in cancer therapy-related pathways.”

In conclusion, we successfully extracted plasma exosomes, and detected the relative expression of miRNA in plasma exosomes by qRT-PCR, demonstrated that hsa-miR-619-5p and hsa-miR-4454 were significantly overexpressed in plasma exosomes in lung adenocarcinoma patients. Therefore, these two genes can be used as markers of lung adenocarcinoma. This work has certain potential in screening miRNAs in exosomes as tumor markers.

## Data Availability

The datasets presented in this study can be found in online repositories. The names of the repository/repositories and accession number(s) can be found in the article/Supplementary Material.
