# Supplementary material for: Identification of hsa-miR-619-5p and hsa-miR-4454 in plasma-derived exosomes as a potential biomarker for lung adenocarcinoma
Source: Front Genet. 2023 May 11;14:1138230. doi: 10.3389/fgene.2023.1138230 (PMC10213947; doi:10.3389/fgene.2023.1138230)
Supplement: Supplementary file 1 [file Table1.DOCX]

STable1. Clinical information of plasma samples

|  | Testing set  （N=6） |  | Validation set  （N=80） |
| --- | --- | --- | --- |
| Categories  Control  Tumor  Gender  Male  Female  Age  ≥50  <50  TNM stage  Ⅰ—Ⅱ  Ⅲ—Ⅳ | 3  3  4  2  6  0  3  0 |  | 40  40  36  44    61  19  34  6 |

STable2. Reverse transcription（RT）primers , forward and reverse primers

| gene | RT primer | Forward primer and  Reverse primer |
| --- | --- | --- |
| hsa-miR-619-5p | GTCGTATCCAGTGCAGGGTCCGAGGTATTCGCACTGGATACGACGGCTCA | GCTGGGATTACAGGCATGAGCC  CAGTGCAGGGTCCGAGGTATTC |
| hsa-miR-4454 | GTCGTATCCAGTGCAGGGTCCGAGGTATTCGCACTGGATACGACTGGTGG | GATCCGAGTCACGGCACCAA  CAGTGCAGGGTCCGAGGTATTC |
